# A qualitative study exploring the perceptions of health among pre‐teen girls from disadvantaged communities in Dublin

**DOI:** 10.1111/chso.12614

**Published:** 2022-07-24

**Authors:** Mckenzie Dow, Celine Murrin, Grace O'Malley, Aoife Brinkley, Silvia Bel‐Serrat

**Affiliations:** ^1^ National Nutrition Surveillance Centre, School of Public Health, Physiotherapy and Sports Science University College Dublin Dublin Ireland; ^2^ W82GO Child and Adolescent Weight Management Service Children's Health Ireland Dublin Ireland; ^3^ Obesity Research and Care Group, School of Physiotherapy RCSI University of Medicine and Health Sciences Dublin Ireland; ^4^ Childrens Health Ireland at Connolly Dublin Ireland

**Keywords:** being healthy, disadvantaged, girls' health, perceptions of health, socioecological, socioeconomic status

## Abstract

There are disparities in health outcomes between youth from higher and lower socioeconomic backgrounds, and girls are especially vulnerable to changes in health‐related behaviours as they develop. Therefore, this study explored how girls from disadvantaged communities in Dublin, Ireland, make sense of ‘being healthy.’ A phenomenological qualitative design was implemented. Three focus groups were conducted (*n* = 22, 10–12 years) and data were analysed using thematic analysis. Food and physical appearance featured prominently within the girls' definitions of health. Girls and their families from low‐SES backgrounds may experience more difficulties with time scarcity as well as environmental barriers to a healthy lifestyle.

## INTRODUCTION

### Socioeconomic disadvantage and inequalities in energy balance‐related behaviours in children

Energy balance‐related behaviours include dietary consumption, as well as sleep and physical activity, and these behaviours have been linked to overweight and obesity (Hopkins & Blundell, 2016; Nuutinen et al., 2017; te Velde et al., 2012). However, evidence suggests these energy balance‐related behaviours are socially determined (Mantziki et al., 2015; Si Hassen et al., 2016). Children attending Irish schools with a disadvantaged status also have higher rates of overweight and obesity compared to peers attending non‐disadvantaged schools, and adolescents from less affluent backgrounds have poorer health outcomes (Bel‐Serrat et al., 2018; World Health Organization. Regional Office for Europe, 2016; Keane et al., 2012). Quantitative studies of families in Ireland indicate that youth from families with less parental education and lower occupation status also have a higher prevalence of overweight and obesity (Walsh & Cullinan, 2015) and similar patterns are evident in other high‐income countries (Vazquez & Cubbin, 2020). Despite the body of quantitative evidence on socioeconomic status (SES) and childhood obesity, less is known about the perspectives on health and weight‐related behaviours from children and adolescents who live in disadvantaged communities. Understanding the perspectives on health from these young people is important for gaining insight into how relevant behaviours might effectively be addressed through changes to public policy.

In a qualitative study used to inform the development of obesity policy in Ireland, consultations with youth were conducted using methods such as world café workshops and body maps to identify variables related to health. The children and adolescents identified variables such as healthy eating, getting enough sleep, exercising regularly, body image and self‐concept, as well as having a support system of family and friends. Furthermore, the community and school settings of participants were identified as being important for maintaining a healthy lifestyle (Martin et al., 2018). In another qualitative study with Irish children, diet was found to be influenced by personal preference, family and school environments, as well as peers (Fitzgerald et al., 2010), but qualitative evidence on health‐related behaviours with a focus on disadvantaged populations is still missing. It is apparent that the concept of ‘being healthy’ during childhood is likely nested in levels of the environment, communities and social circles which have been organised using the socioecological model (SEM) (Casey et al., 2009; Määttä et al., 2016). One example is the DONE framework, created with evidence from several European countries and which outlines several social determinants of nutrition outcomes for children across four levels; individual, interpersonal, environment and policy (Stok et al., 2017). This framework highlights how eating behaviours and nutrition outcomes are strongly socially determined, with a wide array of factors related to eating behaviours being included such as food knowledge, family structure, household socioeconomic status, food availability and accessibility and industry influences.

### Girls' perceptions of health

The transition from childhood to adolescence brings with it more autonomy and new experiences in different environments which can lead to changes in physical activity, social spaces, as well as emotional changes and new ideas about the self. Females experience this transition differently to males, in terms of sex‐determined biological changes and psycho‐social changes (Mendle et al., 2019). Pre‐adolescent girls from disadvantaged communities are also particularly vulnerable to a poorer diet (Tate et al., 2015) and reduced physical activity (Mota et al., 2011) as they develop, with girls consistently participating in lower levels of activity compared to boys (Steene‐Johannessen et al., 2020). This may be as a result of conditions such as being unable to spend as much time outdoors due to living conditions, family difficulties with affording nutritious foods, or prevalence of energy‐dense food outlets in low‐SES communities (Hanson & Chen, 2007). Girls are also particularly vulnerable to messages about body image, weight and dieting (Spencer et al., 2015). Notably, the connections between self‐identity and obesity in adolescence have mainly been investigated in connection to disordered eating and body image (Bray et al., 2018) rather than focusing on socioeconomic or community factors that may shape personal experiences and identity. Therefore, the aim of this study was to understand how girls from disadvantaged communities in Dublin make sense of ‘being healthy’.

## METHODS

This study implemented a qualitative design informed by a social constructivist paradigm, emphasising that individual meaning stems from cultural, societal and environmental constructs, in addition to life experiences (Amineh & Asl, 2015). The study used a phenomenological approach as the research objectives focused on the participants' lived experiences in relation to concepts of health. Previous work has highlighted the value of phenomenological approaches for contributing to a better understanding of what childhood is like in today's world (Danaher & Briod, 2005), particularly for those children who are disenfranchised (Lewthwaite et al., 2017) and who come from disadvantaged circumstances (Andresen & Meiland, 2019). At the time of data collection and analysis, the lead author was a PhD researcher with a background in psychology and public health. The second analyst was a research scientist with a background in nutrition and dietetics and a PhD in nutritional epidemiology. Ethical approval for this study was awarded by the researchers' university Human Research Ethics Committee (LS‐18‐69).

### Participants

Pre‐adolescent females (hereafter ‘the girls’), aged 10–12 years from disadvantaged communities participated in the study (*n* = 22). Disadvantaged communities were identified using the Irish Pobal Index Map of Deprivation (Haase & Pratschke, 2016) for recruitment in youth centres and community outlets. The Pobal Index score is calculated using demographic, social composition and labour market data from the Irish 2016 Census. In addition, freely available information provided by the State listing of primary schools with disadvantaged status was used to recruit participants from schools in County Dublin. Disadvantaged schools (i.e. DEIS schools, ‘Delivering Equal of Opportunity in Schools’) are defined as those schools at social or economic disadvantage and are identified by the Irish Department of Education and Skills (Department of Education, 2019). Contact was made initially by letter, email and phone with school principals and co‐ordinators of youth organisations and community centres to inform them about the study and to facilitate recruitment of participants. These professionals acted as the liaison between the research team and the study participants and distributed consent forms. No specific criteria were given to the contact persons for selecting participants other than age and gender. Of the 92 DEIS schools contacted, four responded with interest but only two participated. Of the 33 community groups, youth centres and non‐profit centres contacted, seven responded with interest, but only one youth group participated.

### Focus groups

The use of focus groups was preferred over individual interviews as data could be collected from more participants with varying experiences in this way. Furthermore, focus groups can be useful when exploring sensitive topics, as they can facilitate in‐group reflexivity and comfortable conversation (Hennessy & Heary, 2005), as well as reduce possible perceptions of a power imbalance between adult interviewer and child participant (Oliveira, 2011). A topic guide was developed with additional input from a clinical psychologist working in a paediatric clinical obesity service, as well as a research psychologist. A pilot focus group was conducted with a convenience sample of four females in early adolescence. From this pilot group, the topic guide was adapted to minimise stigmatising language with regard to weight and appearance. Data collection took place in three centres: a primary school, a youth organisation and a community centre in disadvantaged areas in Dublin (Ireland) between January and December 2019. Three focus group sessions were conducted and participants within each group were known to each other as classmates and friends. Information sheets about the aims of the study were provided to the pre‐adolescent girls and their caregivers and signed caregiver consent forms and child assent forms were returned to the researchers if both agreed for the child to take part.

All the focus group discussions were moderated by the same researcher while another took field notes. Focus groups took place over a year and were digitally recorded with the consent of the participants. The sessions lasted approximately 1 hour each and were held in a quiet room within each organisation (school, youth centre). A teacher requested to be present during the third focus group discussion which was facilitated within their school, although they did not participate. Each focus group started with an opening activity utilising pictures of celebrities, during which the facilitator proceeded to ask questions from the topic guide (Table S1).

### Data collection and analysis

Interviews were transcribed verbatim by the lead researcher and participants were assigned a participant number which served as their identifier throughout the transcript. Thematic analysis (Braun & Clarke, 2006) was conducted by two analysts and each focus group was read through multiple times, allowing for immersion in the data. The focus group transcriptions were coded separately by each analyst and a debriefing session was held between the researchers where codes were compared and any disagreements were resolved together with consideration of their differing professional backgrounds. Coding and theme creation was conducted without qualitative analysis software which has been supported in previous literature (Bennett et al., 2019). A deeper review of themes was conducted by the lead researcher and discussed once again with the second analyst. The lead researcher developed a preliminary mind map of the themes which was also discussed, (Figure S1) and the themes were labelled and described. Quotes that best represented each theme and subtheme were selected by the first researcher and reviewed by the second researcher.

## RESULTS

Findings were organised across four levels, informed by the socioecological model of health (Bronfenbrenner, 1979; McLeroy et al., 1988) (Figure S1). For the purpose of this study the four levels of the socioecological model were: individual, interpersonal, community and organisational, and the wider societal level. However, it is acknowledged that using four levels diverges from other versions of this model that include five or six levels (Pereira et al., 2019). Seven themes were identified from the three focus groups: (1) Looks tell all, (2) health literacy: salads, gym, sleep, repeat, (3) being healthy with my friends, (4) family as role models, (5) our neighbourhood is unhealthy, (6) we learned it at school and (7) social media and health. Some themes also have subthemes (see Table 1).

**TABLE 1:** Summarises the subthemes identified in the study

Theme	Subtheme	Example quotes
Health literacy: Salads, gym, sleep, repeat	**Weight and health are synonymous**—In each group, there was a slow shift where the terms weight and health increasingly were used interchangeably. Being ‘fat’ or ‘too skinny’ was indicative of poor health.	“She's healthy…’Cause she's not too skinny and she's not too fat.” (P4, Focus Group 2) “…they have like a good weight balance? And that's all I can think of.” (P2, Focus Group 3)
	**We know nutrients**—Fruits and vegetables were discussed, particularly salads. Fast foods identified as being enjoyable, but also unhealthy, along with sugar‐sweetened beverages, sugar in tea, and sugary cereals and breads.	“Yeah like, I think that, well like I, this is my like opinion, I like ham, I'd only have ham once a week ‘cause I think that there's loads of fat ‘n’ all in it and I do not like, and I do not like really have it, and I could have plain I do not really have like loads of toppins ‘cause I think it's just loads to put more sugar into loads of your foods that your eatin’ like toppin more foods, like sugars ‘n’ all into your body.” (P8, Focus Group 1)
	**Food can still be confusing**—This included discussions about whether a potato was a fruit or vegetable and whether lasagne was healthy or unhealthy. One group had difficulties naming the vegetables and foods they had tried at school.	P6: “…I was told to eat, see, we were making a seafood…what are they called again?” P1: “Ooooh fish?” P6: “The lettuce around it uh, and fish inside, and rice.” P2: “No, ehm…” P1: “Eh, sushi?” P2: “Yeah, sushi sushi.” P6: “My‐ my teacher she's from Australia…and she got, she made ehm, sea? What's it called again?” (Conversation from Focus Group 2)
	**Phones are good and bad**—Too much screen time could harm their eyes and was considered addictive by some. Phones were also problematic for sleep at night, but were also a way to keep in touch with family and hang out with friends which was deemed healthy.	“It's not unhealthy but, but to some degree it can stop you from sleeping if you use it before bed” (P2, Focus Group 3) “Sometimes important so you can text your family members ‘cause my uncle, he lives in America, and ehm, he's not from America but he lives in America…” (P6, Focus Group 2) “Like I only follow like me friends and stuff.” (P4, Focus Group 3)
	**Comfortable giving health advice—**Participants made firm assessments of why someone was unhealthy and what actions they should take to become healthier.	“I know this girl… she's so insecure about her body she of her kinda like chubbiness, and I told her ‘cause she gets a curry three nights a week and I told her, I goes, a better lifestyle is, better not, you are better off not getting curries and getting’ like salads and stuff, and eating healthy and like if you do good, get a curry like once a month or something.” (P4, Focus Group 1)
Looks tell all	**Health and the self**—Being healthy was linked to confidence and self‐esteem, and likely influenced by the importance placed on appearance and participation in activities.	“Some of them (unhealthy people) feels happy…I would not feel happy” (P5, Focus Group 2) “I think you need to be more active if you wanna be more happy…because sometimes it can change your mood a lot, because ehm, like all that sugar and stuff can put you down…” (P7, Focus Group 1)
	**It is about a mind‐set**—The individual alone was perceived as being responsible for staying fit and living a healthy lifestyle. It was felt that you simply cannot tell others what to do with their body.	“Yeah yeah, it's important to be healthy. But if people feel like, how they feel in their own body, you cannot really make them change it.” (P6, Focus Group 1) “They can do whatever they want with it and like, that's like my Mam's friend. She does not wanna be skinny, she does not wanna do all this and my mam's like yak have to, like you wanna be skinny, you wanna do all the fun stuff’ and like.” (P4, Focus Group 1)
	**‘Fat’ people cannot…"**—Participants assumed that people with overweight did not go to the gym or engage in other types of physical activity, and had different patterns of eating.	“Yeah well sometimes because like I do dancing and like sometimes year weight can affect how like…you do year dance.” (P7, Focus Group 3) “Sometimes if they are taking part in sport, [unhealthy people] would not be able to play with them ‘cause they have been doin’ sport and they would not…they will not be able to do stuff ‘cause they are fat.” (P1, Focus Group 2)
	**Negative connotations—**Perceptions about people with overweight ranged from being concerned to considering overweight as a cause for shame. Losing weight was often seen as a positive thing.	“I do not think much of them, but, ‘cause it's like their life. But I do like worry a bit.” (P6, Focus Group 3) “I know she's healthy because she used to be really big and then she lost all of the weight.” (P4, Focus Group 2)
	**Cosmetic surgery is bad—**Cosmetic procedures were identified as being unhealthy. Fake tan and wearing makeup was also considered unhealthy.	“I do not think she's healthy because like, she had loads of surgery.” (P5, Focus Group 3) “She gets fake tan which is bad ‘cause it's putting’ all like bacteria…bacteria on your skin and ehm, fake acrylic nails which can harm your nails so…” (P7, Focus Group 1)
Family as role models	**My mam motivates me** *—*The most influential people in the girls social network were their family members, particularly mothers.	“My Mam loves being healthy, and like sometimes my Dad. My‐ my Mam motivates, motivates like us all ‘cause my Dad's like, my Dad goes to the gym as well…like we are always healthy but my Mam motivates me the most.” (P8, Focus Group 1)
	**Belongings Over budget**—Money was not as relevant to whether a family is healthy or not healthy. Rather, access to certain resources, especially time, was more important for living a healthy lifestyle.	“…my parents aren't home so I basically left to feed myself. My‐ we always have breads, cereal but I do not wanna eat sugar and bread all day so I end up going to a store or getting junk food, is not healthier anyway [laughs].” (P2, Focus Group 3)
Our neighbourhood is unhealthy	**Sports and classes—**GAA (Gaelic Athletic Association, describes the sports of Gaelic football, and hurling or camogie) was discussed. Some schools had a strong GAA culture. Many girls were also involved in dance classes in their community.	“Our class is mad about GAA.” (P1, Focus Group 2) “On Saturdays I do dancin’ and then like I go straight from dancing to like a fitness class for like strength in me legs to like do dancin’.” (P7, Focus Group 3)

### Individual level themes

There was general agreement among the girls that being healthy was easy, and that the girls could stay healthy using their knowledge and skills. Personal ambition, choices and motivation played a key role in how the girls felt they could be healthy.

“I think it's important if it's their choice like…you cannot really force

someone to do that. But the thing is, it's you, you can help them, you

can motivate them, but you cannot actually do it for them. They have to do it

themselves. So you have to help them push themselves…” (P1, Focus Group 1)

#### Looks tell all

Perceptions of health were strongly influenced by appearance. Skin, hair and weight or body size were typically used to illustrate a person's level of health. Appearance was deemed a good indicator of health, whether it be from Instagram photos or by interacting with people in their day‐to‐day lives. Cosmetic procedures were also deemed unhealthy (see Table 1).

“If you have like spots, on their face, then they're not drinking water.” (P2, Focus Group 1)

“Oh wait! I think she's healthy because she used to be fat and that means she used to eat loads.” (P1, Group 2)

Throughout the focus groups, weight and health were referred to as though they were synonymous, and the girls felt that overweight indicated poor health. Pejorative beliefs about ‘fat’ people were overtly stated, as well as threaded throughout other comments about appearance and health. There was also an emphasis on healthy people having high self‐esteem, better mood and more confidence.

“Some of them (people with overweight and obesity) feel happy…

I would not feel happy.” (P5, Focus Group 2)

#### Health literacy: Salads, gym, sleep, repeat

Participants spoke freely and confidently about nutrition (See Table 1). There was evidence of a capability to recognise healthy and unhealthy foods and high self‐efficacy for making choices; although their options were not always in line with current health recommendations in Ireland (Department of Health, 2020). They also posited why it was important to eat healthily—in order to maintain a certain weight and keep the body strong.

“Um, it, well it…depends, uh my parents aren't home so I basically left

to feed myself. My‐ we always have breads, cereal but I do not wanna eat

sugar and bread all day so I end up going to a store or getting junk food,

is not healthier anyway.” (P2, Group 3)

“…I eat stuff and I get strong” (P6, Group 2)

However, while much of the girls' knowledge about health‐related behaviours was well‐informed, the sources of their health information contributed to a limited understanding of balanced diets in some cases. For example, salads and ‘greens’ in meals were often portrayed by influencers on social media and television.

“She [celebrity] said on her Snapchat, she actually said she was eat‐ she likes

to eat salad everyday…she says she eats salads so that's healthy” (P1, Group 1)

### Interpersonal level themes

Several important people within the participants' interpersonal network contributed to their understandings of what it meant to be healthy or unhealthy. There was some understanding that if someone was never taught how to eat healthy as a child, then it would be difficult for the person to eat healthily as an adult.

“So then like, now you'll learn like to be healthy so you'll still probably be healthy like when you're older.” (P4, Focus Group 3)

People in their social networks did not just influence the girls' distal ideas of what was healthy. Participant's interpersonal networks also served as social support for healthy energy balance‐related behaviours and support from a friend was seen to make participating in physical activity easier.

#### Being healthy with my friends

Friends were an important part of living a healthy lifestyle (See Table 1) and being social was considered healthy in itself. Offline, playing out with friends and younger family members was an opportunity for healthy behaviours such as physical activity. Equally, online, participants could connect with family and friends, which was considered heathy (see subtheme ‘Phones are good and bad’ in Table 1).

“If you are healthy you would usually have more energy, which make you

upbeat which would change how you hang out with friends…”

(P2, Focus Group 2)

“But if you had a, like a healthy friend, like she could, or he could be like

c'mon let us go to gym and, c'mon let us eat this salad and we can go into

Chopped (salads take‐away)…I'll see you tomorrow for your daily jog

around the block.” (P1, Focus Group 1)

#### Family as role models

Parents, siblings and cousins were most often their point of reference for conceptualising healthy and unhealthy people. Many of the activities they deemed to be healthy, such as going to the gym or eating fruits, were carried out with family members. Parents could help them eat healthily by providing ‘nice dinners’, which were defined by the girls as those providing homemade chips and plenty of vegetables rather than the premade dinners or fast food.

“Sometimes, like when my, like my Mam goes over to the gym and my

brothers playin’ football downstairs, like upstairs is a gym and me and

my Mam sometimes we just do like, eh like, we might go on the treadmill

or the cross‐trainer.” (P8, Focus Group 1)

Within the family context, the availability of time was more important than money or belongings in order to maintain a healthy lifestyle (See Table 1). Being healthy was typically a family effort with all family members getting involved and sharing the responsibility. In particular, mothers were role models for health and gatekeepers of energy‐related behaviours, and the importance of having enough time for healthier options was expressed.

“Eh, like some people just like cook processed, processed foods ‘cause they

do not have the time to like make good dinners. But my Mam, like, at,

during the weekdays like she kinda has, like she eh, cooks processed foods

‘cause like we had to get like, I have dance ‘n’ all, and I, we'd a be rushin’

around…” (P3, Focus Group 1)

“…and like, my Mam helps me be healthy ‘cause like…’cause like, like I

eat less junk food when she's in the house. Like when she's in work I like crisps.”

(P4, Focus Group 3)

### Community‐level themes

#### Our neighbourhood is unhealthy

Many participants mentioned various antisocial challenges in their estates which they believed could impact their health, including burning cars or rubbish, as well as the noise at night which prevented them from getting adequate sleep. Living in an apartment block made it difficult to be healthy because there was not enough space to run around, which prompted creative ways to exercise such as creating obstacle courses indoors. Having parks nearby meant they could play and local stables were an opportunity to work with horses and be physically active.

“…it will be hard [to be healthy]… Especially if you live in an

apartment or do not have a backyard.” (P2, Focus Group 3)

“…I'll ride the horses or either I'll groom them and stuff, or either

I'll bring eh, I'll bring little cousins around in the carriage…”.

(P4, Focus Group 1)

#### Learning about health at school

The Irish healthy eating initiative *Food Dudes* (Horne et al., 2009), a programme to promote fruit and vegetable intake among school‐aged children, was frequently mentioned by the girls and resulted in both positive and negative experiences. Learning about healthy food was not always appreciated; they did not always like the healthy food on offer in school and did not enjoy being told what to do.

“…they are like controlling our body. They, you like, ‘eat this, it's very

healthy’, do this, do that. I get annoyed.” (P6, Focus Group 2)

### Wider societal level themes

#### Social media and health

Policy level socioecological determinants did not arise from the focus groups spontaneously. However, social media was a place where most participants were exposed to imagery and information related to health. While it appeared that participants could be misled by imagery and information on social media, they demonstrated the ability to think critically about the information they were presented with online.

“[Female celebrity] looks like she was workin’ out.” (P4, Focus Group 2)

“Um, like about how, how any of the TikTok‐ers, like, look healthy.

Like by, like by doing little adventures. Like I saw this video where they

were doin’ like bike riding on mountains and everything…”.

(P1, Focus Group 3)

“No because, it's just like, no it's behind screens it could be lies. And some

people hide behind screens and be tellin’ lies so I do not really, really trust social media.” (P7, Focus Group 1)

### Overarching theme: Tendency for contradiction

Certain behaviours were deemed unhealthy by the participants, but they engaged in these behaviours anyway. Generally, when the girls noticed others being unhealthy, that was perceived as unacceptable, but they also described engaging in those behaviours themselves. In adulthood this theme would be a reflection of cognitive dissonance, defined as ‘the state of having inconsistent thoughts, beliefs, or attitudes, especially as relating to behavioural decisions and attitude change’.(Harmon‐Jones & Mills, 2019)

“…you are so young that [social media] could put a bad influence on younger

kids…they want to get their lips done or they want to get their butt injections

or, their breast implants and they wanna do all these things but they are only

kids and it's just a bad bad influence. But I still love her anyways. She's pretty.”

(P1, Focus Group 1)

“It's unhealthy, but if you, want to go on it, just go on it.” (P3, Focus Group 2)

## DISCUSSION

This study aimed to understand how girls from disadvantaged communities in Dublin make sense of ‘being healthy’. To the girls, ‘being healthy’ was eating a healthy diet, going to the gym and avoiding cosmetic procedures that changed one's appearance. The participants' interactions with their environment informed their ideas about health and weight, while their environments prompted both positive and negative health‐related behaviours. This process aligns with the construct of reciprocal determinism in social cognitive theory, where individuals, environment and behaviours interact with one another (Baranowski, 1990; Motl, 2007). Participants described how their families modelled healthy behaviours, including healthy eating and exercise and this likely influenced their own health‐related behaviours. The results are similar to those in the Irish study by Fitzgerald et al. (2010) where it was highlighted how children's personal food choices and environmental factors such as the school food environment were interrelated, likely in a reciprocal way.

A tendency for contradicting themselves regarding health‐related behaviours was an overarching theme throughout the focus groups. Previous literature on self‐contradiction is largely focused on adults and cognitive dissonance in relation to smoking (Fotuhi et al., 2013) and less is known about contradictory beliefs in relation to children's health. While the girls had nuanced health knowledge, their ideas about health did not necessarily translate to healthier behaviours; possibly due to additional environmental factors such as food marketing (Cairns et al., 2013; Tatlow‐Golden et al., 2021), local fast food density (Davis & Carpenter, 2009) or the lack of time they described. Generally, children from low‐SES backgrounds are less likely to have dietary intake in line with health recommendations (Hardy et al., 2017; Spence et al., 2018) highlighting how socioeconomic variables may be responsible for disrupting the connection between girls' health literacy and health‐related behaviours. This tendency for contradiction is one area that could be researched further for informing and improving preventative health initiatives with pre‐adolescents.

### Health in the individual context

Some participants wanted to be healthy because they valued health overall. They expressed a self‐efficacy and a sense of personal responsibility in relation to staying healthy and maintaining a healthy weight. One study suggests that children in the United Kingdom take some responsibility for their own health while also recognising that societal power structures and schooling has an effect on health behaviours (Goldthorpe et al., 2019). For many participants, motivation to maintain a healthy lifestyle stemmed from desire to have a nice appearance or to maintain thinness. While sport and exercise were important to the girls, they also focused on dietary intake in relation to their ideas about health and weight. This is significant given that girls who have thin ideals may be at more risk of developing disordered eating behaviours and show more concern with their own weight and body shape (Schuck et al., 2018; Stice & Shaw, 2002). In the present study, overweight was perceived as negative, and weight status was equated to health. Other studies have reported similar findings, where appearance, including body shape and size, are conflated with health (Burrows & Wright, 2004). Notably, when asked directly, some participants described that body size was not relevant to a person's worth, but would then indirectly perpetuate negative ideas about people who were not thin. This is in line with other literature that suggests school‐aged children (like many adults) have implicit weight bias (Harrison et al., 2016), which is particularly important to note given that preliminary evidence suggests that the experience of weight stigma is linked to poorer health behaviours in adults (Lee et al., 2021).

### Health in the interpersonal context

The role of the mother in the development of health literacy and engagement in health behaviours is evident in previous literature (Coto et al., 2019; Hamilton et al., 2020), and was evident in the present study. Personal experiences, cultural backgrounds and values also have a role in parents' abilities to understand nutrition and physical activity guidance (Lovell, 2016). In a sample of dietary gatekeepers, confidence in cooking skills and nutrition knowledge was linked to the consumption of healthy foods and the creation of healthy meals within the family (Reid et al., 2015). In Ireland, parental obesity is a known predictor of childhood obesity, in addition to low maternal education (Keane et al., 2012).

### Community and environmental contexts for health

There was active participation in community activities such as working with horses, going to dance classes, playing traditional Irish sports and going to the gym. This finding suggests there may be other factors that protect girls from sport and exercise dropout which require further investigation. The girls' neighbourhood conditions also had implications for their ability to be physically active in the form of walks and playing out and about with their peers. This finding concurs with previous literature which suggests that certain neighbourhood characteristics are more conducive to positive health outcomes. Perceived local living conditions have also been associated with obesity and sport activity in children (Nogueira et al., 2013) and neighbourhood socioeconomic position has been inversely linked to walkability and access to greenspace (Jacobs et al., 2021). Nonetheless, community lifestyle initiatives and sport‐based interventions may be effective in increasing physical activity in marginalised children (Rosso & McGrath, 2016), but interventions delivered in the school setting may not have an effect (Breslin et al., 2019). More research is therefore needed.

### Findings unique to low‐SES girls

Resources related to socioeconomic status, such as time and belongings, were described by some participants as being more important to health than income (described in the subtheme Belongs over Budget) and the impact of time scarcity and limited resources on health‐related behaviours was highlighted by the girls. These findings demonstrated some experiences that may differentiate low‐SES girls from their higher‐SES peers with regards to perceptions of healthy lifestyles, and previous quantitative literature has supported this finding. For example, in a longitudinal study of Australian adults, both time scarcity and income scarcity were linked to decreased physical activity and increased consumption of less nutritious foods (Venn & Strazdins, 2017). Mothers' time scarcity has also been linked to child health as an important factor in patterns of food consumption and choice (Jabs et al., 2007; Jabs & Devine, 2006; Venn & Strazdins, 2017).

### Implications for research and practice

The results of this study regarding scarcity of time, as well as the neighbourhood conditions have implications for the development of nutritional and healthy lifestyle programming for families from disadvantaged backgrounds. It is possible that interventions tailored for these conditions may be more effective, but more evidence is needed from programmes tailoring their studies to conditions and experiences that are unique to girls from low socioeconomic backgrounds. This is particularly important given evidence that suggests childhood disadvantage can have lasting effects on health through adulthood (Ferraro et al., 2016). The review from Smith, Goss, Issartel and Belton (Smith et al., 2021) highlighted that successful health interventions with adolescents from disadvantaged backgrounds involved hands‐on approaches, peer‐led strategies and focused on more than the individual education component of the socioecological model; also targeting school environments or involving family members. Interventions with girls from disadvantaged backgrounds should be specific to their needs and go beyond the individual components of health, given the significance of wider interpersonal and community‐level factors demonstrated in the present study.

### Strengths and limitations

A strength of this study was the opportunity to conduct a pilot focus group which allowed for practicing focus group facilitation and refinement of the topic guide. Participants from the focus groups also came from varying communities in Dublin, with each group taking place in a different locality. This allowed for a wider range of perspectives and experiences to be explored, although the lack of representation of children from rural communities is a limitation. A further strength is that the same interviewer conducted all three focus groups, as well as the transcription of the digitally recorded data, which allowed for consistency in the delivery of interviewer questions across each group and a true immersion into the data while transcribing and coding. In addition, having a second coder increased the credibility of the findings and the collaboration between the lead researcher and colleagues allowed for triangulation through each phase of the study. While it is important to try to collect enough data to gather as many perspectives from the population as possible, this is not always achievable. Significant resources are required to reach children from disadvantaged backgrounds, and this study was limited in how much and for how long participants could be recruited. Moreover, potential bias was introduced into the sample when principals and other contact persons selected who consent forms were distributed to.

## CONCLUSION

The pre‐adolescent girls from the disadvantaged communities recruited to this study had complex, nuanced and somewhat firm beliefs about what is healthy. Their definitions of being healthy or unhealthy are shaped by family and formed in their community contexts. Appearance was also significant to the girls' understanding of what it meant to be healthy. This finding is important for understanding the motivations for positive health‐related behaviours, and is particularly imperative when developing healthy interventions that are sensitive to concerns about body image and physical appearance. Family members were role models and were highly influential in the girls' perceptions of what it means to be healthy, suggesting that family or caregiver focused interventions could be particularly successful with this population. Notably, time scarcity was linked to the ability of caregivers to help the girls engage in ‘healthy behaviours’. Creating supportive environments also appears to be fundamental in supporting positive health behaviours in this group of female adolescents. Future research should explore the perceptions of health in participants from a range of socioeconomic backgrounds and older age groups.

## FUNDING INFORMATION

The study was supported by the University College Dublin [grant number SF1648] and the Health Research Board Centre for Health and Diet Research. The funding sources had no involvement in the study design, in the collection, analysis and interpretation of data, in the writing of the report, or in the decision to submit the article for publication.

## CONFLICT OF INTEREST

The authors have no conflict of interest to report.

## ETHICS APPROVAL

Ethical approval for this study was awarded by the University's Human Research Ethics Committee (LS‐18‐69).

## PATIENT CONSENT STATEMENT

Consent was granted via consent forms distributed to participants' caregivers. Assent forms were also distributed to the children who participated.

## Supporting information


Appendix S1

## Data Availability

Data from this study are unable to be shared as consent was not received from participants to share the transcribed data collected in the focus groups for other projects.
