# Supplementary material for: A qualitative study exploring the perceptions of health among pre‐teen girls from disadvantaged communities in Dublin
Source: Child Soc. 2022 Jul 24;37(2):579–97. doi: 10.1111/chso.12614 (PMC10087344; doi:10.1111/chso.12614)
Supplement: Supplementary file 1 — Appendix S1 [file CHSO-37-579-s001.docx]

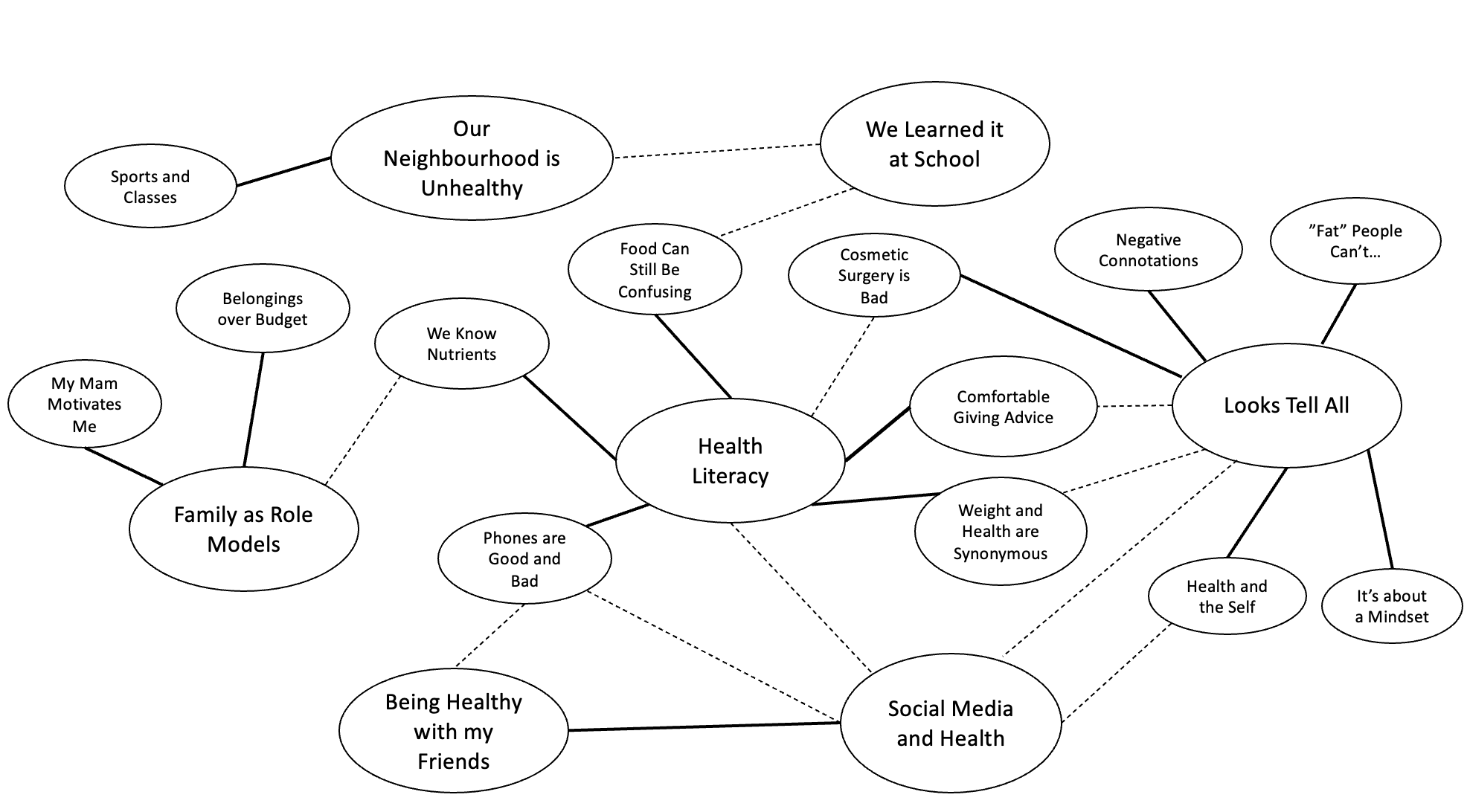
Supplementary Figure 1

Supplementary Figure 2


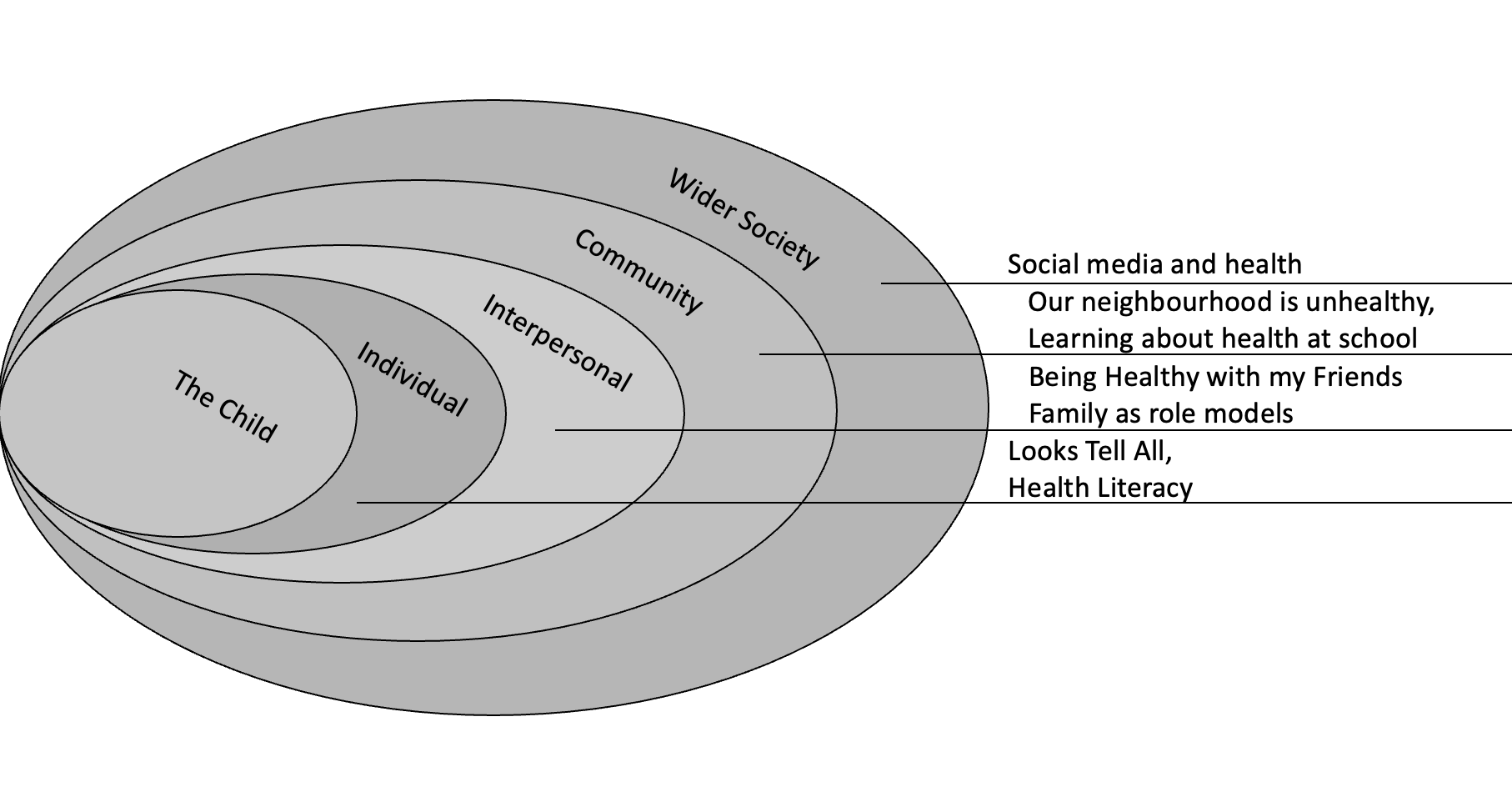


Supplementary Table 1

*Topic Guide (Supplementary Materials)*

| Introduction | Prompts |
| --- | --- |
| Introductions  Opportunity to ask interviewer questions  Reminder of confidentiality and rules | Names  Why the interviewer is there  What you say is private, not telling your parents or teachers [etc.] No right or wrong answers  Be kind and respectful to one another, and try to listen to one another and take turns speaking |
| “Being healthy” | Prompts |
| Conceptualizations of “being healthy” and healthy lifestyles  Language  Social media pictures (3 pictures)  Being unhealthy and feelings, self-esteem | What does “being healthy” mean? To you?  How did you come up with that?  What does a healthy person look like?  What does an unhealthy person look like?  How do you think a healthy person might feel about themselves? Unhealthy person?  Do you being healthy or unhealthy relates to hanging out with friends?  Do you think being unhealthy can affect your moods? How so? |
| Healthy lifestyles | Prompts |
| “Being Healthy”  Importance of Healthy Lifestyles  Unhealthy and feelings | Do you think everyone understands how to be healthy?  Do you think “being healthy” is important right now? Why do you think that?  Think of someone you know who isn’t healthy…what do you think about them? |
| Dietary intake | Prompts |
| Healthy Eating  Unhealthy Eating  Eating Behaviors  Eating behaviours across social settings | What sort of foods are healthy?  Unhealthy?  How much do you think about what you eat? And why is that?  How do you feel about yourself when you eat unhealthy?  Think of someone you know who doesn’t exercise…what do you think of them?  How do you think you can help them?  Is eating healthy easy? Or hard? How come?  Do you ever feel like you don’t want to eat healthy? Why? |
| Physical activity | Prompts |
| Exercise Engagement  Exercise Perceptions  Thoughts about exercising | What types of exercise do you do? Can you tell me about that?  How much do you think about exercising? And why is that?  How did you feel about yourself when you exercise?  Think of someone you know who doesn’t exercise…what do you think of them?  How do you think you can help them?  Is exercising easy? Or hard? How come?  Do you ever feel like you don’t want to exercise? Why? |
| Sleeping habits | Prompts |
| Importance of sleep  Sleeping habits  Barriers to good sleep | How much do you sleep each night?  Do you think you get good sleep?  Have you ever had a time where you didn’t sleep enough?  What are some things that make it hard to have a good sleep? |
| Screen usage | Prompts |
| Types of screen usage/devices  Importance of devices  Devices and Feelings  Devices and “being healthy” | What types of screens or technology do you use the most? [Such as TV, Computer, Video Games, Phones]  When would you use the most?  What do you use your phones [etc.] the most for?  Do you ever stay up too late?  Are your phones important to you?  Do you think using your phones [etc.] is healthy? Unhealthy? |
| Behaviour change | Prompts |
| Explanation of what the researchers want to do  Past experiences with interventions  Perceptions of health interventions | Where do you get information about being healthy? Eating, exercise, sleep etc.  Do you think social media effects the way you think about “being healthy?”  Do you ever see things online that change the way you feel about yourself?  Has anyone else ever talked to you about “being healthy?” Can you tell me about that?  What did you like about that?  What did you dislike?  Do you think helping young people be healthier is something that’s important? Why? Why not?  Who do you think needs the most help being healthier? Why?  If your family started making “healthier changes”, how would you feel?  What about at your school?  If you have a friend who has less money than you, do you think they have a harder time being healthy?  If I wanted to help you be healthier, what’s the best way I can do that? |

*Table S1.* Topic Guide used in Focus Groups with Participants
